# An Analysis for Variable Physical Properties Involved in the Nano-Biofilm Transportation of Sutterby Fluid across Shrinking/Stretching Surface

**DOI:** 10.3390/nano12040599

**Published:** 2022-02-10

**Authors:** Sohaib Abdal, Imran Siddique, Saima Afzal, Somayeh Sharifi, Mehdi Salimi, Ali Ahmadian

**Affiliations:** 1 School of Mathematics, Northwest University, Xi’an 710069, China; sohaib@stumail.nwu.edu.cn; 2 Department of Mathematics, Khawaja Fareed University of Engineering and Information Technology, Rahim Yar Khan 64200, Pakistan; 3 Department of Mathematics, University of Management and Technology, Lahore 54770, Pakistan; imransiddique@umt.edu.pk (I.S.); f2019109028@umt.edu.pk (S.A.); 4 Institut für Numerische Mathematik, Technische Universität Dresden, 01062 Dresden, Germany; somayeh.sharifi@mailbox.tu-dresden.de; 5 Department of Mathematics and Statistics, St. Francis Xavier University, Antigonish, NS B2G 2W5, Canada; 6 Institute of Industry Revolution 4.0, The National University of Malaysia, Bangi 43600, Malaysia; 7 Department of Mathematics, Near East University, Nicosia 99138, Turkey

**Keywords:** sutterby fluid, nanofluid, bioconvection, nano-biofilm stagnation point, magnetohydrodynamic, concentration dependent properties

## Abstract

In this article, we explore how activation energy and varied transit parameters influence the two-dimensional stagnation point motion of nano-biofilm of Sutterby fluids incorporating gyrotactic microbes across a porous straining/shrinking sheet. Prior investigations implied that fluid viscosity as well as thermal conductance are temperature based. This research proposes that fluid viscosity, heat capacity and nanofluid attributes are all modified by solute concentration. According to some empirical research, the viscosity as well as heat conductivity of nanoparticles are highly based on the concentration of nanoparticles instead of only the temperature. The shooting approach with the RK-4 technique is applied to acquire analytical results. We contrast our outcomes with those in the existing research and examine their consistency and reliability. The graphic performance of relevant factors on heat, velocity, density and motile concentration domains are depicted and discussed. The skin friction factor, Nusselt number, Sherwood number and the motile density are determined. As the concentration-dependent properties are updated, the speed, temperature, concentration and motile density profiles are enhanced, but for all concentration-varying factors, other physical quantities deteriorate.

## 1. Introduction

The Sutterby fluid model depicts highly dilute polymer solutions and it is one of the non-Newtonian fluid models used to analyze the rheological properties of various materials. It can anticipate shear thinning and shear thickening properties of a fluid. In squeezing analysis, the Sutterby fluid model is used and the effect of mixed convection on Sutterby fluid is also included in squeezing flow theory. The Sutterby fluid model computes the characteristics of pseudoplastic and as well as dilatant solutions and Sutterby fluid parameters are used to control the flow effectively. Khan et al. [1] examined the radiative, thermophoretic, Brownian motion and stratified features for heat analysis of Sutterby nanofluid employing the Bvp4c technique. Sabir et al. [2] explored the effects of heat radiation as well as an angled magnetic force on the 2-D Sutterby fluid stagnating point motion utilizing the Cattaneo-Christov heat source theory and the Runge–Kutta methodology of fourth order. Ahmad et al. [3] utilized the Homotopy Analysis methodology to investigate the mixed convective features in the squeezed movement of chemically bonded Sutterby fluid in a compressed channel having dual stratification. Refs. [4,5,6] deliberated the distinct flow aspects of Sutterby fluid.

Stagnation-point flow is an important phenomenon since all fluid-flow-solid-structure contact points are related to stagnation-point flow properties. In addition to technological applications, defining the rate of variation of the physical properties around the flow is crucial. The highest heat exchange and pressure, caused by a decrease in velocity, occurs around the surroundings of a flow in stagnation-point flow. Stagnation-point flow has numerous examples in fluid stream and heat exchange in a large variety of manufacturing and engineering fields. Microelectronics thermal structure using fans, thermoplastic extruding, cooling of nuclear plants, doodling of plastic film, wire drawing, heat exchange in atmospheric, heat exchanger, flow-ability, prediction of friction factor difficulties presented in engineering fields and aerodynamic manufacturers are examples of application areas. Such flows are mainly produced by the flowing fluid nearer to the stagnated area of a fixed surface in the fluid or restrained with hydrodynamics. Jusoh et al. [7] employed the finite difference approach to clarify the unstable three-dimensional magneto-hydrodynamic stagnant point motion of nanofluids involving heat generation/absorption. Ghalambaz et al. [8] utilized the Finite-difference with collocation strategy to explore the mixed convective flow as well as heat transmission of an Al${}_{2}$O${}_{3}$-Cu/water hybrid nanofluid across a vertical surface. Abdollahzadeh et al. [9] explored the continuous two-dimensional stagnant point movement of three varieties of nanofluids, specifically Cu-water, Al${}_{2}$O${}_{3}$-water and TiO${}_{2}$-water, against a porous stretched surface utilizing a heat generation, shooting methodology and the fourth-order Runge–Kutta approach. Jafarimoghaddam [10] researched the stagnation-point movement against a porous linearly extending/shrinking wall embedded in copper/water nanofluids employing the Runge–Kutta–Fehlberg Algorithm (RKF45). Refs. [11,12,13] scrutinized the stagnation point flow in various features.

The application of heat transfer determines an important role in magnetic drug targeting, thermal power plants, thermal planning of electronic processes and temperature distribution in tissues and so on. Surface heat transfer rate is greater in the case of hybrid nanofluid than in the case of nanofluid or base liquid. The issue of heat dissipation has become the focal point for ensuring the normal operation of electronic equipment. The inter distribution of heat sources caused by the spatial configuration of elevated components on the modules becomes another problem that needs solving in the heat removal problem of high heat transfer density as the number of electronic equipment components increases. A cooling system’s objective is to keep the energy efficient air frequency inverter pack’s temperature within its operating temperature. Heat generation is considerably large in IGBT modules, IPM modules and other converter components. The component’s heat source discharge cannot fulfil the criteria of semiconductor materials. Makinde et al. [14] analyzed the stable 2-D MHD freely convection boundary-layer motions of an electrical carrying nanofluid across a non-linear extending sheet while accounting for chemical change as well as heat source/sink applying the homotopy analysis approach. Sandeep and Sulochana [15] introduced a unique conceptual model for analyzing the momentum and energy transport performance of Jeffrey, Maxwell as well as Oldroyd-B nanoparticles on an expanding surface in the existence of a transversal magnetic force, a non-uniform heat source/sink, heat radiation and suction impacts. Elgazery [16] addressed the movement of an unstable two-dimensional nanoparticles, consisting of silver Ag, copper Cu, alumina Al${}_{2}$O${}_{3}$ as well as Titania TiO${}_{2}$, across a vertical extending porous surface in the vicinity of an angled magnetic flux and a non-uniform heat generation. Refs. [17,18,19] examined the heat generation in different aspects.

The role of Arrhenius activation energy with binary chemical changes in combination with heat mass transit is critical. Explicitly, the emergence of finite Arrhenius activation energy in an oil field combined with a binary chemically reaction mixture system, material degeneration, analytical chemistry, geological engineering, water and oil emulsification and mechano-chemistry. In general, mass transportation in conjunction with chemical changes is more complicated, as evidenced by the manufacturing and exhaustion of gaseous reactants. Activation energy is the minimum energy needed for the transformation of reaction mixture into products throughout a chemical process. Ahmad and Khan [20] explored the importance of activation energy in the formation of a chemically bond formation while the movement of Sisko fluid across a permeable curving movable surface. Uddin et al. [21] analyzed the chemically reactive nonlinear mixed convection MHD movement of Prandtl–Eyring nanoparticles in the vicinity of activation energy as well as Joule heating. Bhatti et al. [22] examined the influence of activation energy on the movement of gyrotactic microbes in a nanofluid across an extended surface, keeping in mind the cumulative impact of magneto-hydrodynamics and permeability. Refs. [23,24,25] deliberated the implications of activation energy on nanofluids.

Microorganisms are the unique cellular kinds that can be detected in animals, living beings and plants. The irregular movement of microbes in fluids generates bio-convection. Such gyrotactic organisms are substantially thicker than water flowing upward. Two cutaways were inserted in the top frames for quicker access to the upper frets. Two cutaways were incorporated in the lower bouts for convenient access to the highest frets. One more fascinating research region is the bioconvection of nanoparticles, which has a wide range of applications such as microfluidic devices, pharmaceutical production, gas containing, sediment waterways, modeling, fuel, lubrication components, microbial enhanced oil recovery, hydrodynamics scheme, hydrodynamics construction, polymer fabrication, and so on. Habib et al. [26] analyzed slip impacts as well as the effects of activation energy and thermal radiation on MHD nanoparticles in the vicinity of an electromagnetic force and gyrotactic microbes employing the shooting approach. Farooq et al. [27] explored the attributes of bioconvection in Carreau nano-fluid stream under the implications of distinct thermal circumstances along an extended cylinder exhibiting Cattaneo-Christov mass and energy flux. Muhammad et al. [28] examined the time-dependent movement of a magnetically rheological Carreau nanofluid transporting microorganisms across a rotating wedge exhibiting velocity slip as well as heat radiation attributes. Refs. [29,30,31] scrutinized the influence of bioconvection on nanofluids.

Amirsom et al. [32] evaluated the three-dimensional stable stagnant point stream of a bionanofluid exhibiting variable transit parameters reliant on density, zero mass flow rate, and heat convective boundary conditions employing the Runge–Kutta-Fehlberg fourth-fifth order numerical approach (RKF45). In this manuscript, the activation energy as well as transport features of a magnetically nano-biofilm of Sutterby fluids comprising gyrotactic microorganisms across a permeable stretching sheet is analyzed. The mathematical model is tackled numerically by employing RK-4 approach via shooting strategy. Furthermore, the influences of physical parameters are depicted graphically.

## 2. Physical Model and Mathematical Formulation

**Figure 1:**
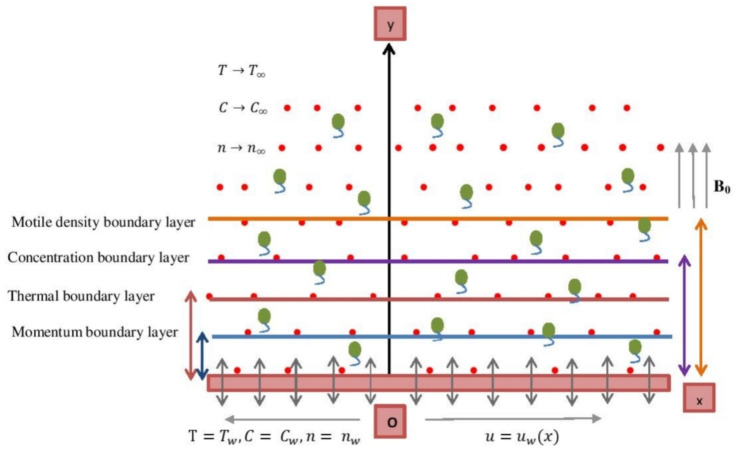
Flowchart of the elongated surface.

We addressed the steady, two-dimensional as well as incompressible flow of Sutterby nanofluid mixed with spherical nanomaterials (that do not swap) with gyrotactic microbes. Heat generation and chemical conversion with activation energy are also illustrated through a prolonging/dwindling sheet. The elongated surface is expected to be permeable, and the stream reaches the region y≥0 with a fixed stationary point at x=0, as seen in Figure 1. We anticipated that at the surface, $T_{w}$ is a consistent heat, $C_{w}$ is the density and $n_{w}$ is the motile microbe concentration, where $T_{\infty }$, $C_{\infty }$ and $n_{\infty }$ are the free stream temperature, intensity and motile microbe density, respectively. Buongiorno’s two-component nanoscale notion is applied as well as sphere nanomaterials inside diluting nanoparticles.

The exterior flowing and extending sheet velocities are specified by $u_{e}\left(x\right)=ax^{m}$ and $u_{w}\left(x\right)=cx^{m}$, respectively, where *a*, *m* as well as *c* are constants while *x* is the external component with a>0 and m>0. The coefficient *m* designates the power-law frequency exponent; m=1 implies a linear state, but m>1 suggests a non-linear one. The criteria m=0 states that the sheet can not elongate or collapse, revealing that it is a fixed sheet. It is indeed noteworthy to observe that c>0 and c<0 describe growing and contracting sheets, respectively. We presume that the varying magnetic field B(x) and variable permeability k(x) do have the forms $B\left(x\right)=B_{0}x^{\frac{m-1}{2}}$ and $k\left(x\right)=k_{0}x^{1-m}$. Many researchers, such as [33,34], have assumed this form of B(x) and k(x).

Assuming such considerations, the relevant mathematical expressions that initiated the current flow conditions can be expressed as follows [35,36]:

(1) $$\frac{\partial u}{\partial x}+\frac{\partial v}{\partial y}=0,$$

$$u\frac{\partial u}{\partial x}+v\frac{\partial u}{\partial y}=u_{e}\frac{du_{e}}{dx}+\left(\frac{1}{\rho }\right)\frac{\partial }{\partial y}\left[\mu_{s}\left(C\right)\frac{\partial u}{\partial y}\right]+\frac{\nu }{2}\frac{\partial^{2}u}{\partial y^{2}}\left(1+\frac{S_{b}b^{2}}{2}{\left(\frac{\partial u}{\partial y}\right)}^{2}\right)-\left(\frac{\sigma^{*}B{\left(x\right)}^{2}}{\rho }+\frac{v}{k(x)}\right)\left(u-u_{e}\right)$$

(2) $$+\left(\frac{1}{\rho }\right)\left[\left(1-C_{\infty }\right)\rho_{\beta }\left(T-T_{\infty }\right)\right]-\left(\rho_{p}-\rho_{f}\right)g\left(C-C_{\infty }\right)-\left(n-n_{\infty }\right)g\gamma \left(\rho_{m}-\rho \right)],$$

(3) $$u\frac{\partial T}{\partial x}+v\frac{\partial T}{\partial y}=\frac{1}{\rho C_{p}}\frac{\partial }{\partial y}\left[k_{s}\left(C\right)\frac{\partial T}{\partial y}\right]+\tau \frac{\partial }{\partial y}\left[D_{B}\left(C\right)\left(C-C_{\infty }\right)\right]\frac{\partial T}{\partial y}-\tau \frac{D_{T}}{T_{\infty }}{\left(\frac{\partial T}{\partial y}\right)}^{2}+Q_{1}\left(T-T_{\infty }\right),$$

(4) $$u\frac{\partial C}{\partial x}+v\frac{\partial C}{\partial y}=\frac{\partial }{\partial y}\left[D_{B}\left(C\right)\frac{\partial C}{\partial y}\right]+\frac{D_{T}}{T_{\infty }}\frac{\partial^{2}T}{\partial y^{2}}-{\left(Kr\right)}^{2}\left(C-C_{\infty }\right){\left(\frac{T}{T_{\infty }}\right)}^{m_{1}}exp\left(\frac{-E_{a}}{k_{2}T}\right),$$

(5) $$u\frac{\partial n}{\partial x}+v\frac{\partial n}{\partial y}=\frac{\partial }{\partial y}\left[D_{n}\left(C\right)\frac{\partial n}{\partial y}\right]-\frac{b_{1}W_{c}}{\Delta C_{w}}\left[\frac{\partial }{\partial y}\left(n\frac{\partial C}{\partial y}\right)\right].$$

$$u=u_{w}\left(x\right)=cx^{m},v=v_{w}\left(x\right)=-\frac{m+1}{2}\sqrt{\frac{u_{e}\left(x\right)\nu }{x}}R,\;T=T_{f},\;C=C_{w},\;n=n_{w},\;\;at\;y=0,$$

(6) $$u\to \;u_{e}=ax^{m},T\to \;T_{\infty },C\to \;C_{\infty },n\to \;n_{\infty },\;\;as\;y\to \infty .$$

In the preceding equations, ν signifies kinematic viscosity, μ specifies stable dynamic viscosity and $C_{p}$ denotes the appropriate temperature at static pressure. $b^{2}$ indicates the consistency index, $\mu_{s}\left(C\right)$ depicts variable dynamic viscosity, $S_{b}$ is the flow deportment index and *m* is the power factor of straining/dwindling velocity. The varying heat conductance is indicated by $k_{s}\left(C\right)$, $b_{1}$ is a chemotactic variable, $W_{c}$ is the optimal cell swimming frequency and $D_{B}\left(C\right)$ symbolizes the varied mass diffusivity of nanoparticles (changing Brownian diffusion variable), $D_{n}\left(C\right)$ is the varied permeability of gyrotactic microorganisms and $D_{T}$ indicates the thermophoretic dispersion variable. $\rho_{\infty }$ depicts the consistent fluid density, $D_{B,\infty }$ represents the static nano-particle mass permeability, and $D_{n,\infty }$ indicates the constant microbial diffusivity. $\tau =\frac{{\left(\rho c\right)}_{p}}{{\left(\rho c\right)}_{f}}$ is the fraction of the effective heat potential of a nanoparticle item to the heat prospect of a base fluid (water), $c_{2}$ is the non-dimensional viscous parameter, $c_{4}$ is the heat conduction factor, $c_{6}$ is the mass diffusion variable and $c_{8}$ is the microbe permeability factor.

## 3. Solution Evaluation

Now, we suggest dimensionless variables which will be described as:

(7) $$\eta =y\sqrt{\frac{u_{e}\left(x\right)}{\nu x}},\;\psi =\sqrt{\left(u_{e}\left(x\right)\nu x\right)}f\left(\eta \right),\;\;\theta \left(\eta \right)=\frac{T-T_{\infty }}{T_{f}-T_{\infty }},\;\;\phi \left(\eta \right)=\frac{C-C_{\infty }}{C_{w}-C_{\infty }},\;\;\chi \left(\eta \right)=\frac{n-n_{\infty }}{n_{w}-n_{\infty }}.$$

Hence, η is the similarity parameter, ψ(x,y) is the stream factor as well as the non-dimensional variables of linear velocity, energy, nanoparticle volume fraction density and motile intensity are f(η), θ(η), ϕ(η) and χ(η), respectively. The stream operator is designed in such a way that *u* and *v* satisfy the continuity condition. The velocity coefficients *u* and *v* are now expressed as:

$$u=\frac{\partial \psi }{\partial y},\;\;\;\;\;\;\;\;\;\;\;\;v=-\frac{\partial \psi }{\partial x}$$

The preceding concentration-dependent characteristics was anticipated by Amirsom et al. [32]:

(8) $$\mu_{s}\left(C\right)=\mu_{\infty }\left[1+c_{1}\left(C-C_{\infty }\right)\right]=\mu_{\infty }c_{2}\phi \left(\eta \right)+\mu_{\infty },$$

(9) $$k_{s}\left(C\right)=k_{\infty }\left[1+c_{3}\left(C-C_{\infty }\right)\right]=k_{\infty }c_{4}\phi \left(\eta \right)+k_{\infty },$$

(10) $$D_{B}\left(C\right)=D_{B,\infty }\left[1+c_{5}\left(C-C_{\infty }\right)\right]=D_{B,\infty }c_{6}\phi \left(\eta \right)+D_{B,\infty },$$

(11) $$D_{n}\left(C\right)=D_{n,\infty }\left[1+c_{7}\left(C-C_{\infty }\right)\right]=D_{n,\infty }c_{8}\phi \left(\eta \right)+D_{n,\infty }.$$

The continuous expression in (1) is effectively satisfied and the non-dimensional versions of Equations (2)–(5) are as follows:

(12) $$\left[\left(1+c_{2}\phi \right)+\frac{S_{b}}{4}R_{es}D_{es}f^{\prime \prime 2}+\frac{1}{2}\right]f^{\prime \prime \prime }+c_{2}\phi^{\prime }f^{\prime \prime }+\frac{m+1}{2}ff^{\prime \prime }-mf^{\prime 2}+\left(M+Kp\right)\left(1-f^{\prime }\right)+\omega \left(\theta -Nr\phi -Rb\chi \right)+m=0,$$

(13) $$\left(1+c_{4}\phi \right)\theta^{\prime \prime }+Pr\frac{m+1}{2}f\theta^{\prime }+c_{4}\theta^{\prime }\phi^{\prime }+Nb\left(1+2c_{6}\phi \right)\theta^{\prime }\phi^{\prime }+Nt\theta^{\prime 2}+Q\theta =0,$$

(14) $$\left(1+c_{6}\phi \right)\phi^{\prime \prime }+Le\frac{m+1}{2}f\phi^{\prime }+c_{6}\phi^{\prime 2}+\frac{Nt}{Nb}\theta^{\prime \prime }-LeC_{1}\phi {\left(1+\delta \theta \right)}^{m^{*}}\exp\left(\frac{-E}{1+\delta \theta }\right)=0,$$

(15) $$\left(1+c_{8}\phi \right)\chi^{\prime \prime }+Sc\frac{m+1}{2}f\chi^{\prime }+c_{8}\phi^{\prime }\chi^{\prime }-Pe\left[\phi^{\prime }\chi^{\prime }+\phi^{\prime \prime }\left(\sigma +\chi \right)\right]=0.$$

as well as the non-dimensional boundary situations (6) that are related with them are:

$$f\left(0\right)=R,\;f^{\prime }\left(0\right)=\lambda ,\;\theta \left(0\right)=1,\;\phi \left(0\right)=1,\;\chi \left(0\right)=1,$$

(16) $$f^{\prime }\left(\infty \right)\to 1,\;\theta \left(\infty \right)\to 0,\;\phi \left(\infty \right)\to 0,\;\chi \left(\infty \right)\to 0.$$

$\lambda =\frac{c}{a}$ represents the expanding (λ>0) or collapsing (λ<0) parameter, and *R* specifies the speed of wall permeability (lateral mass variation), with R>0 for sucked and R<0 for injection. The following parameters of non-dimensional speed, temperature, nanoscale and gyrotactic microbes are:

$$\begin{array}{l} R_{es}=\frac{a}{\nu }x^{3m-1},\;D_{es}=a^{2}b^{2},\;M=\frac{\sigma B_{0}^{2}}{a\rho },\;Kp=\frac{v}{ak_{1}},\;\omega =\frac{\beta g\left(1-C_{\infty }\right)\left(T_{f}-T_{\infty }\right)}{\rho a^{2}x^{2m-1}},\;Nr=\frac{\left(\rho_{p}-\rho \right)\left(C_{w}-C_{\infty }\right)}{\beta \rho \left(1-C_{\infty }\right)\left(T_{f}-T_{\infty }\right)},\\ Rb=\frac{\gamma^{*}\left(n_{w}-n_{\infty }\right)\left(\rho_{m}-\rho \right)}{\beta \rho \left(1-C_{\infty }\right)\left(T_{f}-T_{\infty }\right)},\;C_{1}=\frac{{\left(Kr\right)}^{2}}{ax^{m-1}},\;E=\frac{E_{a}}{K_{p}T_{\infty }},\;Pr=\frac{\nu }{\alpha },\;Q=\frac{Q_{1}}{ax^{m-1}},\;Le=\frac{\nu }{D_{B,\infty }},\\ Nb=\frac{\tau D_{B}\left(C_{w}-C_{\infty }\right)}{\alpha }=\frac{\tau D_{B}\Delta C_{w}}{\alpha },\;Nt=\frac{\tau D_{T}\left(T_{f}-T_{\infty }\right)}{\alpha T_{\infty }}=\frac{\tau D_{T}\Delta T_{f}}{\alpha T_{\infty }},\;Sc=\frac{v_{\infty }}{D_{n,\infty }},\;Pe=\frac{b_{1}W_{c}}{D_{n,\infty }},\\ \delta =\frac{T_{f}-T_{\infty }}{T_{\infty }},\;\sigma =\frac{n_{\infty }}{n_{w}-n_{\infty }}=\frac{n_{\infty }}{\Delta n_{w}},\;\lambda =\frac{c}{a} \end{array}$$

## 4. Physical Quantities

As an engineering aspect, the physical characteristics are massively crucial. These quantities represent the flow features, heat transit rate, mass transfer rate and motile microorganism flux, that are designated as follows:

### 4.1. Skin Friction Coefficient

The surface drag force is computed as follows:

$$Cf_{x}=\frac{\tau_{w}}{\rho_{\infty }u_{e}^{2}}$$

here, $\tau_{w}$ is needed to compute shear force and is denoted as:

$$\tau_{w}=-\mu_{s}\left(C\right)\left[\left(1+\lambda \right)\frac{\partial u}{\partial y}+\frac{S_{b}b^{2}}{3}{\left(\frac{\partial u}{\partial y}\right)}^{3}\right]\;\;\;\;at\;\;y=0$$

Equations (7) and (8) are being used to develop a non-dimensional form of the current equation

$$Cf_{x}{\left(Re_{x}\right)}^{\frac{1}{2}}=-\left(1+c_{2}\phi \left(0\right)\right)\left[\left(1+\lambda \right)f^{\prime \prime }\left(0\right)+\frac{S_{b}}{3}R_{es}D_{es}{\left(f^{\prime \prime }\left(0\right)\right)}^{3}\right]$$

### 4.2. Nusselt Number

The preceding is the mathematical representation of this relationship between heat transport reliability:

$$Nu_{x}=\frac{xq_{w}}{k_{s}\left(C\right)\left(T_{f}-T_{\infty }\right)}$$

where, the exterior heat gradient is represented by the symbol $q_{w}$ and is specified as follows:

$$q_{w}=-k_{s}\left(C\right)\frac{\partial T}{\partial y}\;\;\;\;at\;\;y=0$$

Equations (7) and (9) are employed to modify the above equation as:

$$Nu_{x}{\left(Re_{x}\right)}^{-1/2}=-\theta^{\prime }\left(0\right)$$

### 4.3. Sherwood Number

The mass transit rate coefficient is mathematically explored as follows:

$$Sh_{x}=\frac{xq_{m}}{D_{B}\left(C\right)\left(C_{w}-C_{\infty }\right)}$$

here, $q_{m}$ signifies surface mass variation, that is specified as:

$$q_{m}=-D_{B}\left(C\right)\frac{\partial C}{\partial y}\;\;\;\;at\;\;y=0$$

Utilizing Equations (7) and (10), the non-dimensional representation of the above equation is as follows:

$$Sh_{x}{\left(Re_{x}\right)}^{-1/2}=-\phi^{\prime }\left(0\right)$$

### 4.4. Density of Micro-Organisms

The density of miroorganisms is presented as follows:

(17) $$Nn_{x}=\frac{xq_{n}}{D_{n}\left(C\right)\left(n-n_{\infty }\right)}$$

here, $q_{n}$ describes motile microorganism transmission and is written as:

(18) $$q_{n}=-D_{n}\left(C\right)\frac{\partial n}{\partial y}\;\;\;\;at\;\;y=0$$

The non-dimensional composition of the expression is as below, employing Equations (7) and (11).

$$Nn_{x}{\left(Re_{x}\right)}^{\frac{-1}{2}}=-\chi^{\prime }\left(0\right)$$

where,

$$Re_{x}=\frac{xu_{e}\left(x\right)}{v_{\infty }}\;\;\;\;is\;the\;Reynolds\;quantity.$$

## 5. Solution Procedure

It is obvious that the specified problems (12)–(15) considering boundary restrictions (16) could not be resolved mathematically owing to their appreciable non-linearity. As a response, we apply the well-known shooting strategy to approximate the numerical results where the first boundary value problem is transformed into an initial value problem by applying the preceding presumptions. The solution to the significantly nonlinear transformed boundary values problem (12)–(15) is as follows [37,38,39]:

$$\begin{array}{l} S_{1}^{\prime }=S_{2}\\ S_{2}^{\prime }=S_{3}\\ S_{3}^{\prime }=\frac{-1}{\left[\left(1+c_{2}S_{6}\right)+\frac{S_{b}}{4}R_{es}D_{es}S_{3}^{2}+\frac{1}{2}\right]}\left[c_{2}S_{3}S_{7}+\frac{m+1}{2}S_{1}S_{3}-mS_{2}^{2}+\left(M+Kp\right)\left(1-S_{2}\right)+\omega \left(S_{4}-NrS_{6}-RbS_{8}\right)+m\right]\\ S_{4}^{\prime }=S_{5}\\ S_{5}^{\prime }=\frac{-1}{\left(1+c_{4}S_{6}\right)}\left[Pr\frac{m+1}{2}SS_{5}+c_{4}S_{5}S_{7}+Nb\left(1+2c_{6}S_{6}\right)S_{5}S_{7}+NtS_{5}^{2}+QS_{4}\right]\\ S_{6}^{\prime }=S_{7}\\ S_{7}^{\prime }=\frac{-1}{\left(1+c_{6}S_{6}\right)}\left[Le\frac{m+1}{2}SS_{7}+c_{6}S_{7}^{2}+\frac{Nt}{Nb}S_{5}^{\prime }-LeC_{1}S_{6}{\left(1+\delta S_{4}\right)}^{m^{*}}\exp\left(\frac{-E}{1+\delta S_{4}}\right)\right]\\ S_{8}^{\prime }=S_{9}\\ S_{9}^{\prime }=\frac{-1}{\left(1+c_{8}S_{6}\right)}\left[Sc\frac{m+1}{2}S_{1}S_{9}+c_{8}S_{7}S_{9}-Pe\left[S_{7}S_{9}+S_{7}^{\prime }\left(\sigma +S_{8}\right)\right]\right] \end{array}$$

along with the boundary conditions:

$$\begin{array}{l} S_{1}=R,\;S_{2}=\lambda ,\;S_{4}=1,\;S_{6}=1,\;S_{8}=1\;\;at\;\;\eta =0\\ S_{2}\to 1,\;S_{4}\to 0,\;S_{6}\to 0,\;S_{8}\to 0\;as\;\eta \to \infty \end{array}$$

The existence of exponentially convergence is established for $\eta_{max}=5$. All numeric values derived in this situation are restricted to a $10^{-5}$ range.

## 6. Analysis of Results

This segment focused on the rheological attributes of regulating factors on non-dimensional velocity $f^{\prime }\left(\eta \right)$, temperature gradient θ(η), concentration pattern ϕ(η) as well as motile microbe profile χ(η). To this end, relevant parameters such as power law exponent *m*, variable viscosity $c_{2}$, heat capacity $c_{4}$, mass diffusivity $c_{6}$, species diffusivity $c_{8}$, flow deportment index $S_{b}$, mixed convection factor ω, Sutterby Reynolds number $R_{es}$, Sutterby Deborah number $D_{es}$, bioconvection Rayleigh number Rb, porosity factor Kp, buoyancy ratio variable Nr, magnetic field component *M*, thermophoresis parameter Nt, Brownian motion Nb, activation energy *E*, Lewis number Le, heat source/sink factor *Q*, Peclet number Pe and Schmidt number Sc are all visualized. Physical properties such as the Nusselt number, surface drag coefficient, Sherwood quantity and motile intensity component are tabulated. For computational processes, the fixed values of non-dimensional parameters are given as *M* = 0.5, $S_{b}$ = 0.5, $R_{es}$ = 0.5, $D_{es}$ = 0.5, *m* = 2.0, ω = 0.1, Nr = 1.0, Rb = 1.0, Nb = 0.1, Nt = 0.1, *Q* = 0.3, Le = 4.0, *E* = 0.3, Sc = 3.0, Pe = 0.1, σ = 0.1, $c_{2}$ = 0.4, $c_{4}$ = 0.4, $c_{6}$ = 0.4 and $c_{8}$ = 0.4.

Table 1 compares the coefficients of $f^{\prime \prime }\left(0\right)$, $\theta^{\prime }\left(0\right)$ and $\phi^{\prime }\left(0\right)$ to those recorded by Alsenafi et al. [40] and Zaimi et al. [41]. The comparative analysis was conducted by ignoring the occurrence of gyrotactic microbes (by eliminating Equation (18) and assigning $V_{0}$ = 0 and λ = 1 in the boundary constraints (19)).

### 6.1. Impacts of Distinct Parameters on Physical Quantities

Table 2, Table 3, Table 4 and Table 5 illustrate the implications of varied parameters on the skin friction factor, Nusselt number, Sherwood quantity, and motile density quantity, in that sequence. As per Table 3, raising the amounts of heat capacity $c_{4}$, nanoparticles mass diffusion coefficient $c_{6}$, Brownian motion parameter Nb, thermophoresis factor Nt as well as the source of heat *Q* diminish the efficiency of heat transfer $-\theta^{\prime }\left(0\right)$. Table 4 depicts that the mass transmission rate $\phi^{\prime }\left(0\right)$ advances when the quantities of parameters like Le, Nb as well as Nt expand, whereas it declines in the situation of $c_{6}$ and *E*. Table 5 verifies that Sc, Pe as well as σ boost the motile concentration coefficient $-\chi^{\prime }\left(0\right)$, while $c_{8}$ lowers it.

### 6.2. Influence of Power Law Index m

**Figure 2:**
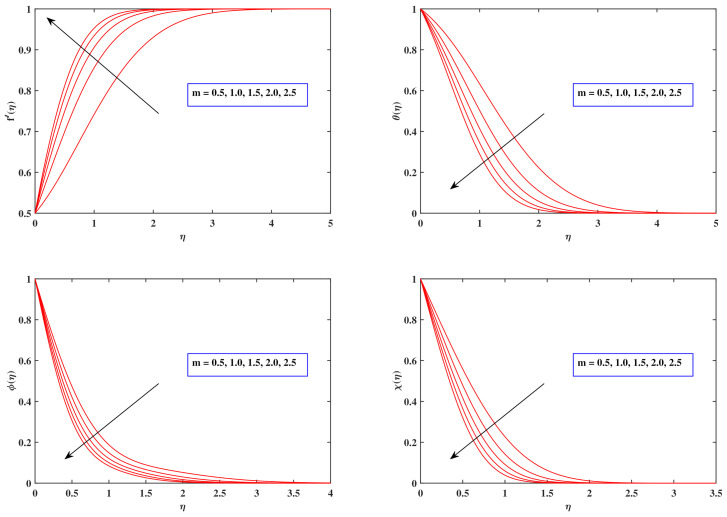
Fluctuation in profiles (velocity, temperature, concentration, and motile density) with *m*.

Figure 2 depicts the impacts of a power law coefficient *m* on velocity, heat, nanoparticle density and motile density variations. While the magnitude of the power law factor *m* develops, the velocity distribution $f^{\prime }\left(\eta \right)$ enhances, whereas the temperature pattern θ(η), density variation ϕ(η) and motile concentration dispersion χ(η) decline.

### 6.3. Effects of Concentration-Dependent Parameters

**Figure 3:**
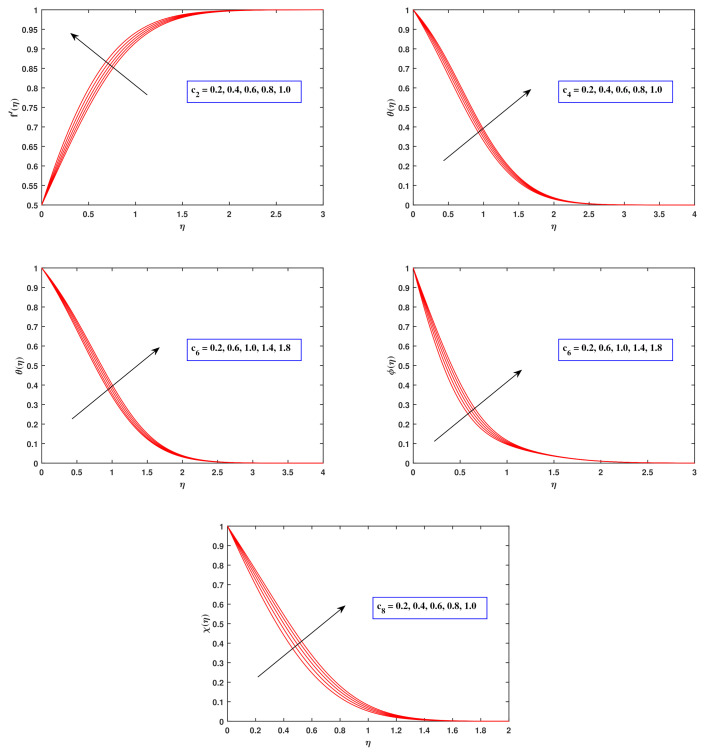
Fluctuation in profiles (velocity, temperature, concentration and motile density) with concentration dependent parameters.

Figure 3 exhibits the implications of a viscosity parameter $c_{2}$, heat diffusivity $c_{4}$, nanoparticle mass diffusing factor $c_{6}$ and microbe diffusivity $c_{8}$ on the velocity, heat, concentration as well as motile density distributions. The graph depicts that as the amount of $c_{2}$ grow, the momentum gradient $f^{\prime }\left(\eta \right)$ tends to arise. It is also worth emphasizing that the heat gradient θ(η) advances as the amount of $c_{4}$ and $c_{6}$ rises. Heat capacity is the tendency to transport energy across a medium, as anticipated. The elevation in thermal transport noted as the quantity of variable heat conductivity is enhanced could be owing in part to the availability of nanoparticles, which have been reported to boost fluid thermal conductance. The concentration of nanoparticles ϕ(η) improves as the quantity of $c_{6}$ climbs. The claim is focused on the concept that fluctuations in nanoparticle concentration influence mass transport. This generates nanoparticle distribution and unconstrained movement that accelerates mass diffusion. While the parameter $c_{8}$ for microorganism species permeability is modified, the numeric estimations of microorganism density χ(η) rise exponentially.

### 6.4. Velocity Profiles

**Figure 4:**
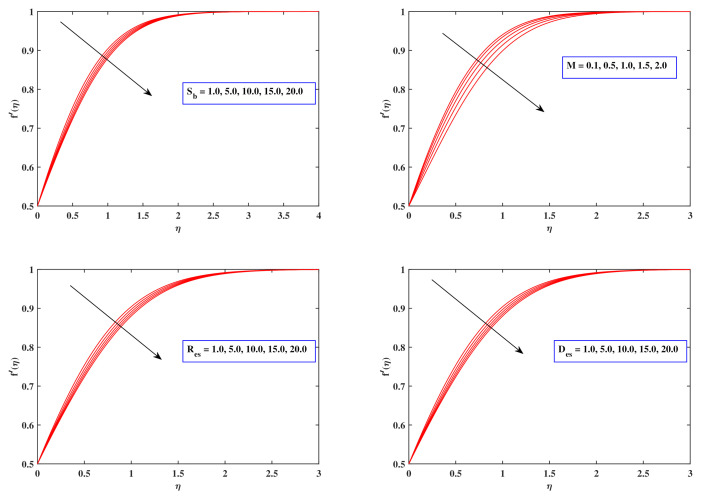
Fluctuation in velocity profile with $S_{b}$, *M*, $R_{es}$ and $D_{es}$.

**Figure 5:**
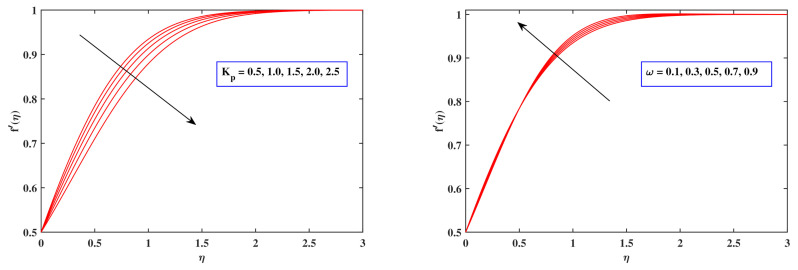

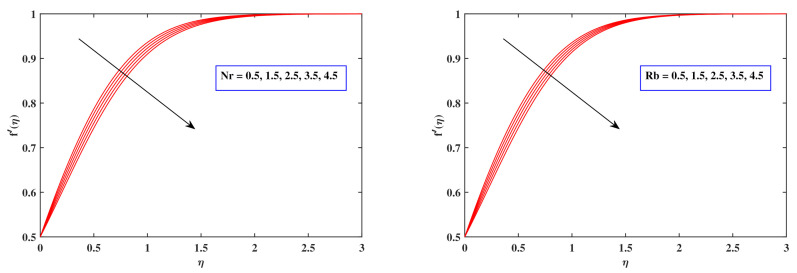
Fluctuation in velocity profile with $K_{p}$, ω, Nr and Rb.

Figure 4 potrays the implications of flow deportment index $S_{b}$, magnetic field parameter *M*, Sutterby Reynolds number $R_{es}$ and Sutterby Deborah number $D_{es}$ on the velocity dispersion $f^{\prime }\left(\eta \right)$. With increasing $S_{b}$, the fluid velocity $f^{\prime }\left(\eta \right)$ deteriorates. In reality, as $S_{b}$ rises, fluid viscosity increases and, since $S_{b}$ grows, opposing forces expand. As a result, the velocity gradient diminishes. As can be evident, raising the amount of *M* minimizes the velocity field of the fluid. The velocity distribution diminishes when the drag force improves owing to progressively expanding amounts of the magnetic flux parameter *M*. The Deborah number is specified by the proportion of indicative time to distortion time frame. The Deborah factor is employed to indicate the object’s viscoelastic property. The higher the Deborah value, the more rigid the substance is; the lower the Deborah number, the better fluid it is. The graph vividly reveals that boosting the Deborah number $D_{es}$ results in a reduction in flowing fluid. As an outcome, the velocity distribution $f^{\prime }\left(\eta \right)$ diminishes. The relationship between inertia forces and viscosity forces is expressed as the Reynolds factor. As the Reynolds number increases, the liquid grows extra sticky. The viscous effects exceed the inertial energies, leading to the flow rate slowing down. The implications of the porous parameter Kp, a mixed convection factor ω, the buoyancy ratio parameter Nr as well as the Rayleigh number Rb upon the velocity variation $f^{\prime }\left(\eta \right)$ are illustrated in Figure 5. The velocity drops as the amount of Kp rises. It is commonly termed as a permeability medium, so it pertains to the penetrating of a permeable material that passively prohibits fluid particles from moving through. It is also accepted that as the value of a mixed convection parameter ω improves, so too does the velocity. Raising the mixed convection factor ω permits the buoyant force to overwhelm the inertia force, yielding a dominant surge of $f^{\prime }\left(\eta \right)$. A reduction in the flow velocity is evident for growing amounts of both Nr and Rb. The fundamental reason for such a propensity may be related to the premise that both the buoyant ratio parameter Nr as well as the bioconvection Rayleigh variable Rb incorporate buoyancy effects which enable velocity variance to decline. The buoyant force correlated with motile microbes generates the majority of the variation in Nr.

### 6.5. Temperature Distributions

**Figure 6:**
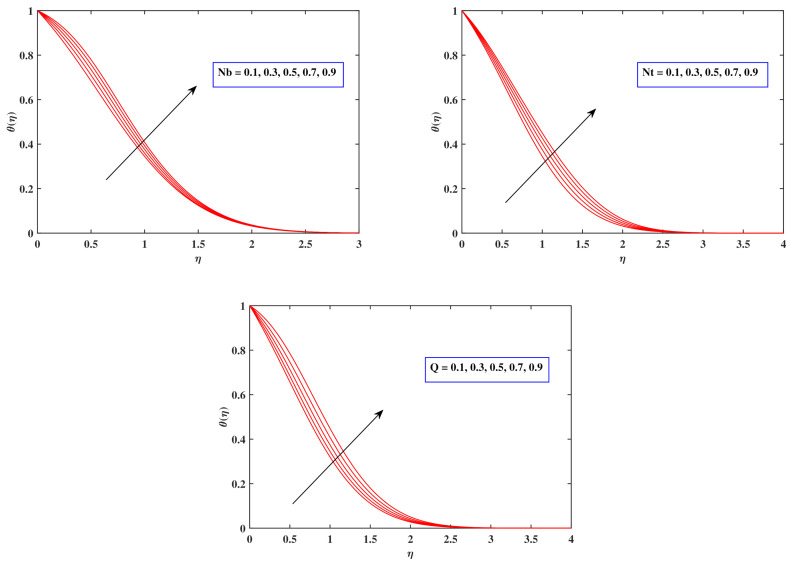
Fluctuation in temperature profile with Nb, Nt and *Q*.

Figure 6 displays the influence of numerous associated flow parameters upon temperature distributions θ(η). It has been observed that as the amount of Nb climbs, so does the temperature of nanoparticles. Temperature distribution developed whenever the Brownian motion factor interacted with the irregular migration of thermal liquid particles. As the quantity of Nt improves, the temperature gradient reveals a progressing pattern. The concept of thermophoresis is very exciting, and it has been used in a diversity of architectural and manufacturing projects. In this procedure, temperature-affected nanoparticles were pulled aside from a hot zone and into a chilly one, enabling the fluid temperature to elevate. As per graph, a significant fluctuation in the heat source variable *Q* emits higher energy into the fluid, resulting in an enhancement in the heat boundary layer depth and temperature pattern.

### 6.6. Concentration Profiles

**Figure 7:**
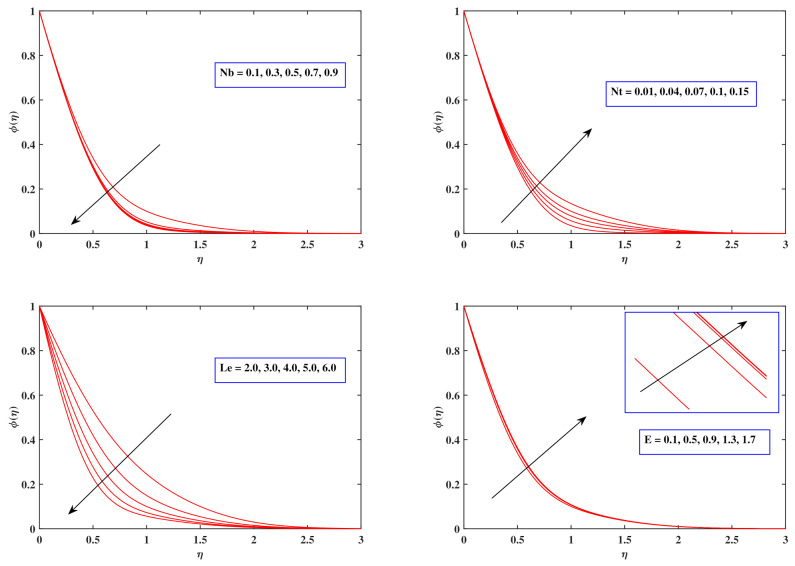
Fluctuation in concentration profile with Nb, Nt, Le and *E*.

Figure 7 potrays the consequences of varied fluid flow factors on the concentration pattern ϕ(η). It depicts the lesser concentration variation generated by increasing the Brownian motion Nb values. The basis of the diminution in the density domain is the correlation of Brownian motion with Brownian diffusion coefficient, which is crucial for diminishing the density field. It was revealed that as the quantity of Nt improves, so too does the concentration of nanoparticles. Thermophoresis is widely noticed in a range of physical scenarios where heat transport is more essential. Due to the higher temperature near the surface, the fluid molecules move to the fairly cooler edge as a response to the thermal disparity, and the density distribution develops as a result. The lower concentration trend generated by Le is related to the presumption that Le is connected directly to the mass diffusion coefficient, which minimizes the density variation. The amount of energy which initiates the reaction method is termed as activation energy. The density dispersion emerges as a response to the interactions of an activation energy parameter *E*.

### 6.7. Motile Density Distributions

**Figure 8:**
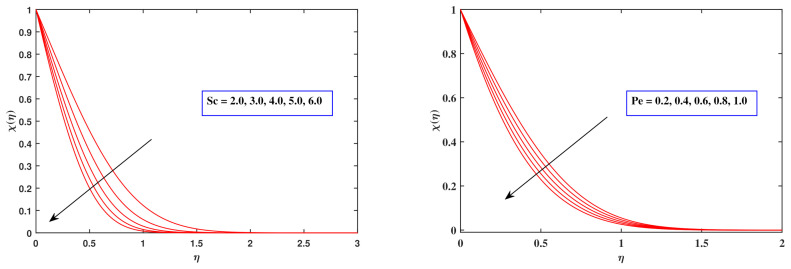
Fluctuation in motile density profile with Sc and Pe.

Figure 7 indicates how the Schmidt number Sc and the Peclet quantity Pe influence the motile density trend χ(η). In an ambiguous way, the Schmidt factor Sc is linked to species diffusing motion. As an outcome, enhancing this factor enables the motile intensity variation to be reduced (Figure 8). Raising Pe displays a progressive pattern over the entire domain. It is due to the premise that Pe exhibits the opposite relationship to the microorganism diffusion coefficient, prompting χ(η) to decline.

## 7. Conclusions

In the proposed inquest, an initiative was conducted to analyze numerous features of 2-D Sutterby fluid-flow across an elongating/shrinking sheet along with stagnant point, magnetic flux as well as concentration-varying aspects. The similarity transformations are employed to modify the controlling PDEs into a system of ODEs. These are then managed by relying on a well-known strategy recognized as the shooting approach, as well as the 4th order Runge–Kutta technique. The following are the major conclusions from the proposed investigation:

- The velocity profile accelerates as the power law coefficient *m* improves, while the heat, density as well as motile concentration trends deteriorate. Variable transiting parameters $c_{2}$, $c_{4}$, $c_{6}$ and $c_{8}$ optimize velocity, heat, density and motile concentration distribution.
- When the factors $S_{b}$, *M*, $R_{es}$, Kp, $D_{es}$, Nr as well as Rb updated, a declining velocity trend is viewed, which is dramatically exaggerated when the ω is inspected.
- Brownian motion parameter Nb, heat conduction factor *Q* as well as thermophoresis parameter Nt all assist towards a relatively consistent temperature variation.
- The nano-particle density pattern strengthens while the activating energy *E* and thermophoresis factor Nt expand, and it swiftly declines as the Lewis number Le as well as the Brownian motion factor Nb develop. item The density of microbes grows as the Schmidt coefficient Sc as well as the Peclet number Pe rise.
- As the amounts of the parameters $c_{4}$, $c_{6}$, Nb, Nt and *Q* expanded, the heat transport capacity declined.
- The Sherwood number drops as the factors *E* and $c_{6}$ expand, but it grows with the variables Le, Nb and Nt boost.
- With growing species dispersion parameter $c_{8}$, microorganism concentration drops swiftly, which is accentuated by raising Schmidt quantity Sc, Peclet number Pe and bioconvection variable σ.

## Figures and Tables

**Table 1:** The comparative outputs.

	Alsenafi et al. [40]	Zaimi et al. [41]	Present Results
$f^{\prime \prime }\left(0\right)$	0	0	0
$-\theta^{\prime }\left(0\right)$	0.476745	0.476737	0.476744
$-\phi^{\prime }\left(0\right)$	1.045230	1.045154	1.04513

**Table 2:** Results for $-f^{\prime \prime }\left(0\right)$.

$c_{2}$	$S_{b}$	$R_{es}$	$D_{es}$	*m*	*M*	ω	Nr	Rb	$-f^{\prime \prime }\left(0\right)$
0.2	0.5	0.5	0.5	2.0	0.5	0.1	1.0	1.0	0.2399
0.4									0.1351
0.6									0.0472
0.4	0.1								0.1322
	0.3								0.1336
	0.5								0.1351
	0.5	0.5							0.1351
		1.0							0.1387
		1.5							0.1423
		0.5	0.5						0.1351
			0.9						0.1380
			1.3						0.1409
			0.5	1.0					0.1003
				2.0					0.1351
				3.0					0.1355
				2.0	0.1				0.1240
					0.3				0.1296
					0.5				0.1351
					0.5	0.1			0.1351
						0.3			0.1282
						0.5			0.1213
						0.1	0.1		0.1411
							0.5		0.1384
							1.0		0.1351
							1.0	0.1	0.1412
								0.5	0.1385
								1.0	0.1351

**Table 3:** Results for $-\theta^{\prime }\left(0\right)$.

$c_{4}$	$c_{6}$	Nb	Nt	*Q*	$-\theta^{\prime }\left(0\right)$
0.2	0.4	0.1	0.1	0.3	0.5670
0.4					0.5074
0.6					0.4605
0.4	0.2				0.5130
	0.4				0.5074
	0.6				0.5018
	0.4	0.1			0.5074
		0.2			0.4573
		0.3			0.4122
		0.1	0.1		0.5074
			0.2		0.4879
			0.3		0.4693
			0.1	0.1	0.5903
				0.2	0.5496
				0.3	0.5074

**Table 4:** Results for $-\phi^{\prime }\left(0\right)$.

$c_{6}$	Le	Nt	Nb	*E*	$-\phi^{\prime }\left(0\right)$
0.2	4.0	0.1	0.1	0.3	1.8349
0.4					1.6460
0.6					1.5017
0.4	3.0				1.3848
	4.0				1.6460
	5.0				1.8812
	4.0	0.1			1.6460
		0.2			1.6511
		0.3			1.6686
		0.1	0.1		1.6460
			0.2		1.6646
			0.3		1.6695
			0.1	0.1	1.6737
				0.2	1.6593
				0.3	1.6460

**Table 5:** Results for $-\chi^{\prime }\left(0\right)$.

$c_{8}$	Sc	Pe	σ	$-\chi^{\prime }\left(0\right)$
0.2	3.0	0.1	0.1	1.5608
0.4				1.3925
0.6				1.2618
0.4	3.0			1.3925
	4.0			1.6249
	5.0			1.8386
	3.0	0.1		1.3925
		0.2		1.4895
		0.3		1.5875
		0.1	0.1	1.3925
			0.3	1.4068
			0.5	1.4211

## Data Availability

Not applicable.

## Nomenclature

**List of Symbols**
u,v	Nanofluid velocity components
(x,y)	Cartesian Coordinates
$u_{w}\left(x\right)$	Stretching/shrinking velocity
$u_{e}\left(x\right)$	Ambient fluid velocity
$v_{w}\left(x\right)$	Wall transpiration velocity
$B_{0}$	Magnetic field strength
*C*	Concentration of nanoparticles
*T*	Temperature of nanoparticles
*n*	Density of micro-organisms
$k_{1}$	Permeability of porous medium
$k_{s}\left(C\right)$	Variable thermal conductivity
$k_{\infty }$	Constant thermal conductivity
$c_{p}$	Specific heat at constant pressure
*g*	Gravitational accerlation
*c*	Constant in stretching/shrinking velocity
$c_{2}$	Viscosity parameter
$c_{4}$	Thermal conductivity parameter
$c_{6}$	Nanoparticle mass diffusivity
$c_{8}$	Micro-organisms species diffusivity
$D_{B}\left(C\right)$	Variable Brownian diffusion coefficient
$D_{T}$	Thermophoresis diffusion coefficient
$D_{n}\left(C\right)$	Variable diffusivity of micro-organisms
$D_{B,\infty }$	Constant nano-particle mass diffusivity
$D_{n,\infty }$	Constant micro-organisms diffusivity
$T_{\infty }$	Uniform temperature in the free stream
$C_{\infty }$	Uniform nanofluid concentration in free stream
$n_{\infty }$	Uniform density of microorganisms in free stream
$Q_{1}$	Heat source/sink coefficient
$E_{a}$	Activation energy
*M*	Magnetic field parameter
Kp	Porosity parameter
Pr	Prandtl number
Nb	Brownian motion parameter
Nt	Thermophoresis parameter
Sc	Schmidt number
*Q*	Heat source parameter
Le	Lewis number
Pe	Peclet number
Nr	Buoyancy ratio parameter
Rb	Rayleigh number
$D_{es}$	Sutterby Deborah number
$R_{es}$	Sutterby Reynolds number
$W_{c}$	Maximum cell swimming speed
$T_{w}$	Uniform temperature at the sheet surface
$C_{w}$	Uniform nanoparticles concentration at sheet surface
$n_{w}$	Uniform density of micro-organisms at sheet surface
*f*	Dimensionless stream function
*a*	Constant in the ambient fluid velocity
*b*	Chemotaxis constant
$m^{*}$	Fitted rate parameter
*R*	Wall transpiration parameter
$S_{b}$	Deportment index flow
$b^{2}$	Consistency index
***Greek Symbols***	
$\alpha_{\infty }$	Uniform thermal diffusivity
$\mu_{\infty }$	Constant dynamic viscosity
$\mu_{s}\left(C\right)$	Variable dynamic viscosity
ν	Kinematic viscosity
σ	Bioconvection constant
$\sigma^{*}$	Electrical conductivity
ρ	Density of fluid
$\rho_{\infty }$	Constant fluid density
$\gamma^{*}$	Average volume of micro-organisms
ω	Mixed convection parameter
λ	Stretching/shrinking parameter
ψ	Dimensionless stream function
η	Dimensionless transverse coordinate
θ	Dimensionless temperature function
ϕ	Dimensionless density of nanoparticles
χ	Dimensionless density of micro-organisms

## References

[1] Khan W., Anjum N., Waqas M., Abbas S., Irfan M., Muhammad T. (2021). Impact of stratification phenomena on a nonlinear radiative flow of sutterby nanofluid. J. Mater. Res. Technol..

[2] Sabir Z., Imran A., Umar M., Zeb M., Shoaib M., Raja M.A.Z. (2021). A numerical approach for 2-D Sutterby fluid-flow bounded at a stagnation point with an inclined magnetic field and thermal radiation impacts. Therm. Sci..

[3] Ahmad S., Farooq M., Javed M., Anjum A. (2018). Double stratification effects in chemically reactive squeezed Sutterby fluid flow with thermal radiation and mixed convection. Results Phys..

[4] Akram J., Akbar N.S., Tripathi D. (2020). Blood-based graphene oxide nanofluid flow through capillary in the presence of electromagnetic fields: A Sutterby fluid model. Microvasc. Res..

[5] Nawaz M. (2020). Role of hybrid nanoparticles in thermal performance of Sutterby fluid, the ethylene glycol. Phys. A Stat. Mech. Its Appl..

[6] Ramesh K., Prakash J. (2019). Thermal analysis for heat transfer enhancement in electroosmosis-modulated peristaltic transport of Sutterby nanofluids in a microfluidic vessel. J. Therm. Anal. Calorim..

[7] Jusoh R., Nazar R., Pop I. (2019). Impact of heat generation/absorption on the unsteady magnetohydrodynamic stagnation point flow and heat transfer of nanofluids. Int. J. Numer. Methods Heat Fluid Flow.

[8] Ghalambaz M., Roşca N.C., Roşca A.V., Pop I. (2019). Mixed convection and stability analysis of stagnation-point boundary layer flow and heat transfer of hybrid nanofluids over a vertical plate. Int. J. Numer. Methods Heat Fluid Flow.

[9] Abdollahzadeh M., Sedighi A.A., Esmailpour M. (2018). Stagnation point flow of nanofluids towards stretching sheet through a porous medium with heat generation. J. Nanofluids.

[10] Jafarimoghaddam A. (2020). Numerical analysis of the nanofluids flow near the stagnation point over a permeable stretching/shrinking wall: A new modeling. Arab. J. Sci. Eng..

[11] Khan M., El Shafey A., Salahuddin T., Khan F. (2020). Chemically Homann stagnation point flow of Carreau fluid. Phys. A Stat. Mech. Its Appl..

[12] Li X., Khan A.U., Khan M.R., Nadeem S., Khan S.U. (2019). Oblique stagnation point flow of nanofluids over stretching/shrinking sheet with Cattaneo–Christov heat flux model: Existence of dual solution. Symmetry.

[13] Arani A.A.A., Aberoumand H. (2021). Stagnation-point flow of Ag-CuO/water hybrid nanofluids over a permeable stretching/shrinking sheet with temporal stability analysis. Powder Technol..

[14] Makinde O.D., Mabood F., Ibrahim M.S. (2018). Chemically reacting on MHD boundary-layer flow of nanofluids over a non-linear stretching sheet with heat source/sink and thermal radiation. Therm. Sci..

[15] Sandeep N., Sulochana C. (2018). Momentum and heat transfer behaviour of Jeffrey, Maxwell and Oldroyd-B nanofluids past a stretching surface with non-uniform heat source/sink. Ain Shams Eng. J..

[16] Elgazery N. (2019). Nanofluids flow over a permeable unsteady stretching surface with non-uniform heat source/sink in the presence of inclined magnetic field. J. Egypt. Math. Soc..

[17] Mebarek-Oudina F. (2019). Convective heat transfer of Titania nanofluids of different base fluids in cylindrical annulus with discrete heat source. Heat Transf. Res..

[18] Upreti H., Pandey A.K., Kumar M., Makinde O. (2020). Ohmic heating and non-uniform heat source/sink roles on 3D Darcy–Forchheimer flow of CNTs nanofluids over a stretching surface. Arab. J. Sci. Eng..

[19] Azizul F.M., Alsabery A.I., Hashim I., Chamkha A.J. (2021). Impact of heat source on combined convection flow inside wavy-walled cavity filled with nanofluids via heatline concept. Appl. Math. Comput..

[20] Ahmad L., Khan M. (2019). Importance of activation energy in development of chemical covalent bonding in flow of Sisko magneto-nanofluids over a porous moving curved surface. Int. J. Hydrog. Energy.

[21] Uddin I., Ullah I., Ali R., Khan I., Nisar K. (2021). Numerical analysis of nonlinear mixed convective MHD chemically reacting flow of Prandtl–Eyring nanofluids in the presence of activation energy and Joule heating. J. Therm. Anal. Calorim..

[22] Bhatti M.M., Shahid A., Abbas T., Alamri S.Z., Ellahi R. (2020). Study of activation energy on the movement of gyrotactic microorganism in a magnetized nanofluids past a porous plate. Processes.

[23] Abdelmalek Z., Khan S.U., Awais M., Mustfa M.S., Tlili I. (2021). Analysis of generalized micropolar nanofluid with swimming of microorganisms over an accelerated surface with activation energy. J. Therm. Anal. Calorim..

[24] Khan W., Ali M., Shahzad M., Sultan F., Irfan M., Asghar Z. (2020). A note on activation energy and magnetic dipole aspects for Cross nanofluid subjected to cylindrical surface. Appl. Nanosci..

[25] Shah Z., Kumam P., Deebani W. (2020). Radiative MHD Casson Nanofluid Flow with Activation energy and chemical reaction over past nonlinearly stretching surface through Entropy generation. Sci. Rep..

[26] Habib D., Salamat N., Abdal S., Siddique I., Ang M.C., Ahmadian A. (2021). On the role of bioconvection and activation energy for time dependent nanofluid slip transpiration due to extending domain in the presence of electric and magnetic fields. Ain Shams Eng. J..

[27] Farooq U., Waqas H., Khan M.I., Khan S.U., Chu Y.M., Kadry S. (2021). Thermally radioactive bioconvection flow of Carreau nanofluid with modified Cattaneo-Christov expressions and exponential space-based heat source. Alex. Eng. J..

[28] Muhammad T., Alamri S.Z., Waqas H., Habib D., Ellahi R. (2021). Bioconvection flow of magnetized Carreau nanofluid under the influence of slip over a wedge with motile microorganisms. J. Therm. Anal. Calorim..

[29] Habib D., Abdal S., Ali R., Baleanu D., Siddique I. (2021). On bioconvection and mass transpiration of micropolar nanofluid dynamics due to an extending surface in existence of thermal radiations. Case Stud. Therm. Eng..

[30] Ahmad F., Gul T., Khan I., Saeed A., Selim M.M., Kumam P., Ali I. (2021). MHD thin film flow of the Oldroyd-B fluid together with bioconvection and activation energy. Case Stud. Therm. Eng..

[31] Song Y.Q., Waqas H., Al-Khaled K., Farooq U., Khan S.U., Khan M.I., Chu Y.M., Qayyum S. (2021). Bioconvection analysis for Sutterby nanofluid over an axially stretched cylinder with melting heat transfer and variable thermal features: A Marangoni and solutal model. Alex. Eng. J..

[32] Amirsom N., Uddin M., Ismail A. (2016). Three dimensional stagnation point flow of bionanofluid with variable transport properties. Alex. Eng. J..

[33] Mabood F., Khan W., Ismail A.M. (2015). MHD boundary layer flow and heat transfer of nanofluids over a nonlinear stretching sheet: A numerical study. J. Magn. Magn. Mater..

[34] Jafar A.B., Shafie S., Ullah I. (2020). MHD radiative nanofluid flow induced by a nonlinear stretching sheet in a porous medium. Heliyon.

[35] Fayyadh M.M., Naganthran K., Basir M.F.M., Hashim I., Roslan R. (2020). Radiative MHD sutterby nanofluid flow past a moving sheet: Scaling group analysis. Mathematics.

[36] Abdal S., Alhumade H., Siddique I., Alam M.M., Ahmad I., Hussain S. (2021). Radiation and Multiple Slip Effects on Magnetohydrodynamic Bioconvection Flow of Micropolar Based Nanofluid over a Stretching Surface. Appl. Sci..

[37] Abdal S., Siddique I., Alshomrani A.S., Jarad F., Din I.S.U., Afzal S. (2021). Significance of chemical reaction with activation energy for Riga wedge flow of tangent hyperbolic nanofluid in existence of heat source. Case Stud. Therm. Eng..

[38] Abdal S., Habib U., Siddique I., Akgül A., Ali B. (2021). Attribution of Multi-slips and Bioconvection for Micropolar Nanofluids Transpiration Through Porous Medium over an Extending Sheet with PST and PHF Conditions. Int. J. Appl. Comput. Math..

[39] Abdal S., Siddique I., Alrowaili D., Al-Mdallal Q., Hussain S. (2022). Exploring the magnetohydrodynamic stretched flow of Williamson Maxwell nanofluid through porous matrix over a permeated sheet with bioconvection and activation energy. Sci. Rep..

[40] Alsenafi A., Bég O.A., Ferdows M., Bég T.A., Kadir A. (2021). Numerical study of nano-biofilm stagnation flow from a nonlinear stretching/shrinking surface with variable nanofluid and bioconvection transport properties. Sci. Rep..

[41] Zaimi K., Ishak A., Pop I. (2014). Stagnation-point flow toward a stretching/shrinking sheet in a nanofluid containing both nanoparticles and gyrotactic microorganisms. J. Heat Transf..

